# Investigation of the Structure-Property Effect of Phosphorus-Containing Polysulfone on Decomposition and Flame Retardant Epoxy Resin Composites

**DOI:** 10.3390/polym11020380

**Published:** 2019-02-21

**Authors:** Wei Zhao, Yongxiang Li, Qiushi Li, Yiliang Wang, Gong Wang

**Affiliations:** 1 CAS Key Laboratory of Space Manufacturing Technology, Technology and Engineering Center for Space Utilization, Chinese Academy of Sciences, Beijing 100094, China; liyongxiang@csu.ac.cn (Y.L.); liqiushi17@csu.ac.cn (Q.L.); 2 University of Chinese Academy of Sciences, Beijing 100049, China; 3 Department of Chemistry and Center for Nano and Micro Mechanics, Tsinghua University, Beijing 100084, China; wangyiliang1988@163.com

**Keywords:** polysulfone, epoxy resin, flame retardant, structure-property effect, kinetics, glass transition temperature

## Abstract

The flame retardant modification of epoxy (EP) is of great signification for aerospace, automotive, marine, and energy industries. In this study, a series of EP composites containing different variations of phosphorus-containing polysulfone (with a phosphorus content of approximately 1.25 wt %) were obtained. The obtained EP/polysulfone composites had a high glass transition temperature (*T*_g_) and high flame retardancy. The influence of phosphorus-containing compounds (ArPN_2_, ArPO_2_, ArOPN_2_ and ArOPO_2_) on the thermal properties and flame retardancy of EP/polysulfone composites was investigated by thermogravimetric analysis (TGA), differential scanning calorimetry (DSC), a UL-94 vertical burning test, and cone calorimeter tests. The phosphorus-containing polysulfone enhanced the thermal stability of EP. The more stable porous char layer, less flammable gases, and a lower apparent activation energy at a high degree of conversion demonstrated the high gas inhibition effect of phosphorus-containing compounds. Moreover, the gas inhibition effect of polysulfone with a P–C bond was more efficient than the polysulfone with a P–O–C bond. The potential for optimizing flame retardancy while maintaining a high *T*_g_ is highlighted in this study. The flame-retardant EP/polysulfone composites with high thermal stability broaden the application field of epoxy.

## 1. Introduction

Epoxy resin (EP) and its composites, as advanced composite materials, are essential for the aerospace, automotive, marine, and energy industries. It is vital to develop the next generation of lightweight, energy-efficient EP composites, owing to their excellent specific stiffness, high strength, and good chemical resistance [1,2]. Applications in the above fields also require EP with high flame retardancy and high continuous service temperature. However, due to the plasticizing effect of flame retardants, the flame retardant modification of EP is very likely to result in a reduction in glass transition temperature (*T*_g_) [3,4]. Hence, the main challenge in enhancing the flame retardancy of EP is to maintain a high glass transition temperature at the same time.

Present technologies for enhancing the flame retardancy of EP rely on the chemical structural modification of EP and the incorporation of flame retardants [5,6]. Unfortunately, the chemical incorporation of phosphorus into epoxide or curing agents always brings undesirable *T*_g_ decreases in the EP composites. An addition of polymeric flame retardant harbors the potential to show no or negligible negative effects on the *T*_g_, which is highly desirable [7]. Several flame retardant elements (phosphorus, nitrogen, sulfur, silicon, etc.) and groups can be incorporated into backbones or side chains of polymeric flame retardants and play a “group synergistic effect” to improve the flame retardancy of EP, such as phosphate [8], phosphonate [9], phosphonamide [10], and phosphazene [11]. Although previous studies have shown that an addition of polysulfone containing phosphate can improve the flame retardant properties of EP, the influence of phosphorus-containing polysulfones on the glass transition temperature and flame retardancy is rarely considered [12,13].

Here, in order to determine the structure–property effect on the flame retardancy and *T*_g_ of EP composites, polysulfone with different phosphorus-containing chemical structures is used to prepare of EP composites. The amount of phosphorus content in the EP/polysulfone composites was restricted to approximately 1.25 wt %. To evaluate the influence of polysulfone with different phosphorus-containing structure, the thermal and flame-retardant properties of EP composites were carefully studied. Moreover, the flame retardant mechanisms of polysulfones in condensed and gas phases were especially investigated.

## 2. Experimental Section

### 2.1. Materials

**Figure 1:**
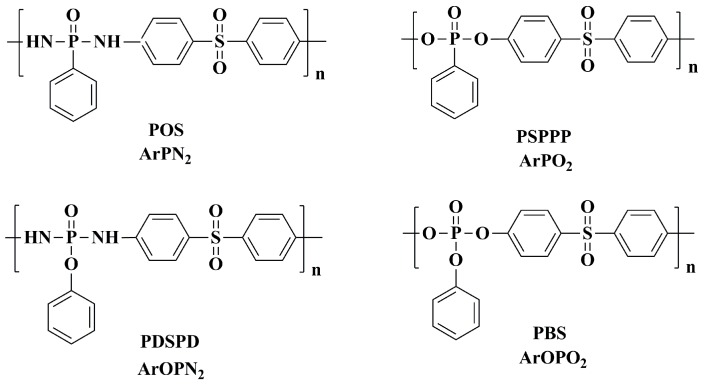
Chemical structures of the polysulfones used in this study.

Epoxy resin (DGEBA, commercial name: E-44) was supplied by Sinopec Baling Company (Yueyang, China). Phenylphosphonic dichloride and phenylphosphate dichloride were purchased from Sigma-Aldrich (St. Louis, MO, USA) and freshly distilled before use. Acetonitrile, methanol, dimethyl sulfoxide, triethylamine, and trichloromethane were purchased from the Beijing Chemical Company, Beijing, China, and used as received. 4,4′-Diaminodiphenyl sulfone, 4,4′-diaminodiphenyl methane, 4,4′-diaminodiphenyl ether, and m-phenylenediamine (m-PDA) were purchased from Sinopharm Chemical Reagent Beijing Co., Ltd., Beijing, China. The poly(4,4′-diamino diphenyl sulfone phenyl phosphonamide) (ArPN_2_), poly(bisphenol sulfone phenyl phosphonate) (ArPO_2_), poly(4,4′-dia-minodiphenyl sulfone phenyl dichlorophosphate) (ArOPN_2_), and poly(bisphenol sulfone phenoxy phosphate) (ArOPO_2_) were synthesized as described in detail by Wang [9], Zhao [10], Liaw [14], and Tai [15] et al. The chemical structure of the polysulfones used is summarized in Figure 1.

### 2.2. Preparation of EP/Polysulfone Composites

Polysulfone powder and DGEBA were mixed in a one-neck flask with a magnetic stirrer to form a solution. Next, m-PDA was dropped into the DGEBA/polysulfone solution. The mixture was placed in a Teflon mold and heated at 80 °C for 2 h and post-cured at 120 °C for 2 h to obtain the EP/polysulfone composites. The amount of polysulfone is listed in Table 1. The phosphorus content of the final epoxy resin was controlled at an overall phosphorus content of approximately 1.25 wt %. The resulting neat epoxy resins are referred to as EP/ArPN_2_, EP/ArPO_2_, EP/ArOPN_2_, and EP/ArOPO_2_. The chemical compositions are summarized in Table 1.

### 2.3. Measurements and Characterization

Thermogravimetric analysis (TGA) was performed with a Mettler-Toledo TGA/DSC-1 thermogravimetric analyzer (Greifensee, Switzerland) under nitrogen at a heating rate of 20 °C/min. About 5.0 mg of the sample was put in an alumina crucible and heated from 50 to 700 °C. A Fourier-transform infrared spectrometer (TGA-FTIR, Nicolet iS10, Waltham, MA, USA) was coupled to the TGA analyzer by a quartz capillary at 300 °C to detect volatile pyrolysis products. Each sample was placed in an alumina crucible and heated from 50 to 600 °C at a heating rate of 20 °C/min under nitrogen. The mass of the sample was about 5 mg in each test.

Differential scanning calorimetry (DSC) was performed with a DSC Q2000 (TA Ltd., New Castle, DE, USA) at a heating rate of 20 °C/min under nitrogen. The glass transition temperatures (*T*_g_) were read at the mid-point of the inflection curve resulting from the typical second heating in the range 40~220 °C.

A standard UL-94 vertical test was carried out on a CZF-3 instrument (Jiangning Analysis Instrument Factory, Nanjing, China) with a sample size of 125 × 12.5 × 3.2 mm^3^ according to ASTM D3081. The burning grade was classified as V-0, V-1, V-2, or fail according to the self-extinguishing time and dripping. Cone calorimeter (CONE) measurements were carried out in a Fire Testing Technology apparatus (East Grinstead, London, UK) at a heat flux of 50 kW/m^2^ according to ISO5660-1. The size of the specimens was 100 × 100 × 1.2 mm^3^. During the test, each specimen was mounted on aluminum foil. All the measurements were repeated three times, and the results were averaged.

X-ray photoelectron spectroscopy (XPS) was determined by a PHI Quantera II SXM (ULVAC-PHI, Inc, Chigasaki, Kanagawa, Japan) at 25 W under a vacuum lower than 10^−6^ Pa. The morphologies of residual char obtained from cone calorimeter tests were observed by a Hitachi S4800 (Hitachi High-Technologies Co., Tokyo, Japan) scanning electron microscope with an acceleration voltage of 15 kV. Each sample was sputtered with a gold layer to ensure surface conductivity.

## 3. Results and Discussion

### 3.1. Thermal Behaviors

**Figure 2:**
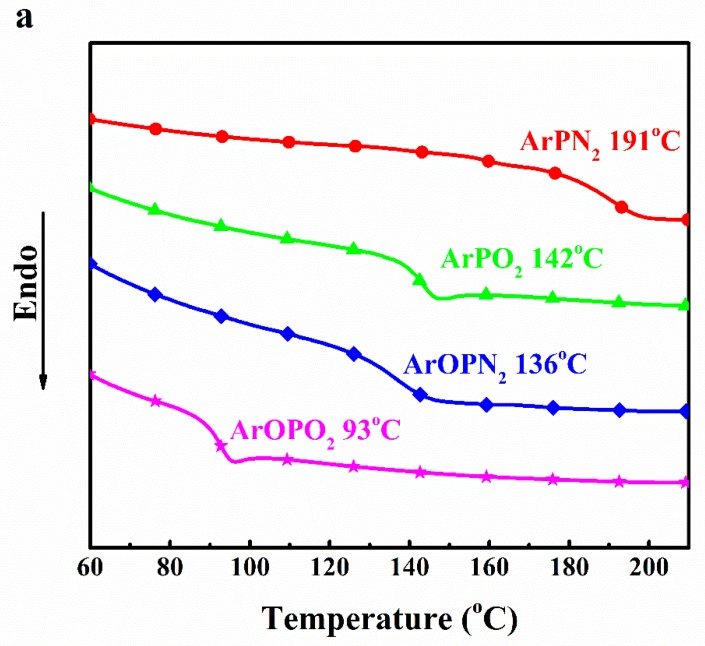

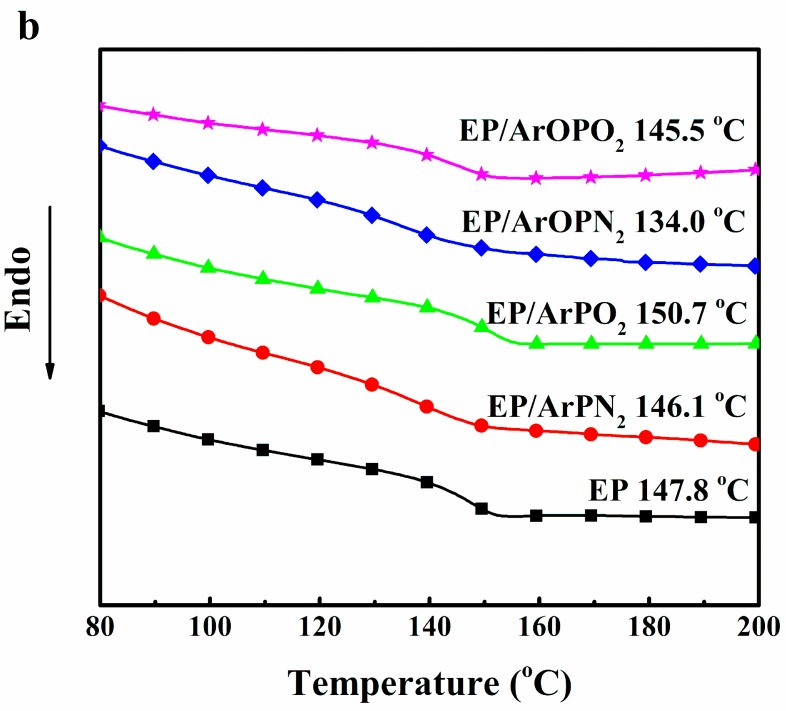
Differential scanning calorimetry (DSC) curves of polysulfone (**a**) as well as EP and EP/polysulfone composites (**b**).

Glass transition temperature (*T*_g_) is one of the most important physical properties of EP and significantly affects the applications of composites. Polymeric flame retardants are preferred to low-molecular flame retardants due to their better compatibility with matrix resin and their resulting in less migration [7]. The glass transition temperature of the polysulfone, EP, and EP/polysulfone composites were investigated by DSC and the main results are further summarized in Table 2. Moreover, the DSC curves of the polysulfones, EP, and EP/polysulfone composites are shown in Figure 2.

All epoxy composites have a close glass transition temperature, suggesting that the addition of polysulfone had no obvious influence on the thermal stability of EP. Although the *T*_g_ values of the polysulfones shows great differences ranging from 93 to 191 °C, only a slight influence of the polysulfones on the *T*_g_ of epoxy resins can be found. This phenomenon agreed with a previous report that polymeric flame retardant can reduce the loss of the thermophysical properties of a resin. The main chain of polysulfone may have a complex interaction with epoxy resin, helping to ensure that EP composites maintain high thermo-properties. The order of the *T*_g_ values for all EP composites were as follows: EP/ArOPN_2_ (134.0 °C) < EP/ArOPO_2_ (145.5 °C) < EP/ArPN_2_ (146.1 °C) < EP (147.8 °C) < EP/ArPO_2_ (150.7 °C). This could be because the P-O-C bond in the side chain improves the flexibility of the polysulfone molecular chain, resulting in a slightly lower crosslinking density in the molecular structure of EP. Polysulfones show great advantages in developing EP composites with a high *T*_g_ value.

**Figure 3:**
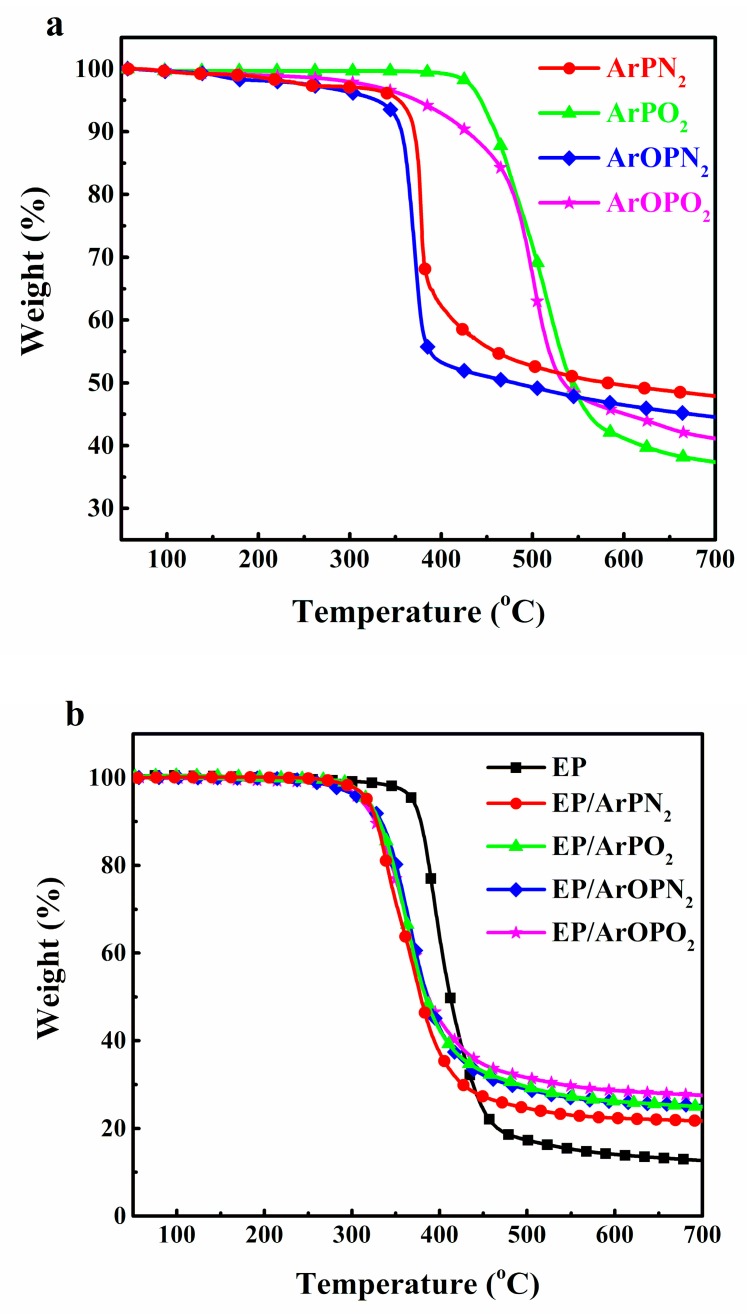
Thermogravimetric analysis (TGA) curves of polysulfone (**a**) as well as EP and EP/polysulfone composites (**b**).

In order to investigate the relationship between the molecular structure of polysulfone and the thermal stability of EP/polysulfone, TGA was used to characterize the thermal stability of polysulfones, EP, and EP/polysulfone composites. Figure 3 shows the TGA and DTG curves of samples under nitrogen, and detailed data are shown in Table 2. TGA curves shown in Figure 3a exhibited a one-step degradation process, and the initial decomposing temperature (the temperature range of a 5% weight loss, *T*_5%_) was concentrated in a high temperature range (330–443 °C), suggesting the good thermal stability of these polysulfones. The *T*_5%_ of ArPN_2_ and ArOPN_2_ was much lower than those of ArPO_2_ and ArOPO_2_, demonstrating that a higher thermal stability of phosphate than phosphamide. Moreover, the thermal degradation of polysulfones led to the formation of an intumescent char layer, which acted as a protective layer for the polymer surface. Therefore, a high residual char yield of polysulfone at 700 °C was obtained. The order was as follows: ArPO_2_ (37.4 wt %) < ArOPO_2_ (41.1 wt %) < ArOPN_2_ (44.5 wt %) < ArPN_2_ (47.9 wt %). The high thermal stability of polysulfones makes it possible to obtain high flame retardancy in EP/polysulfone composites.

In Figure 3b, the *T*_5%_ of the EP/polysulfone composites is shifted to a lower temperature compared with those of pure EP. The residual char yield at 700 °C was much higher than that from EP as well as the calculated value. The order was as follows: EP/ArPN_2_ (21.7 wt %) < EP/ArPO_2_ (24.9 wt %) < EP/ArOPN_2_ (25.2 wt %) < EP/ArOPO_2_ (27.5 wt %). The lower decomposition temperature and higher residual char value can be explained by the catalysis effect of polysulfones on EP. The residual char yield reveals that the char formation effect of polysulfone with a P–O–C bond is more efficient than the polysulfone-containing P–C bond.

All EP/polysulfone composites decomposed at a similar temperature, ranging from 310 to 317 °C, and the maximum weight loss rate occurred at around 365 °C with a 60 wt % mass loss. Thus, phosphorus-containing structures of polysulfones with certain amounts of phosphorus have a slight influence on the thermal decomposition behavior of EP.

### 3.2. Flammability

The flame retardant performance of EP and EP/polysulfone composites was investigated via a UL-94 vertical burning test and CONE calorimeter analysis, and the results are given in Table 1 and Table 3. The order of UL-94 vertical burning test performances of the EP composites (with a phosphorus content of approximately 1.25 wt %) was as follows: EP/ArPN_2_ > EP/ArPO_2_ > EP/ArOPN_2_ ≥ EP/ArOPO_2_. Both EP/ArPN_2_ and EP/ArPO_2_ passed the UL-94 vertical burning test with grades of V-0 and V-1, respectively, whereas the other EP composites had not achieved any grade.

In agreement with previous reports, it was concluded that the ArPN_2_ and ArPO_2_ with a P–C bond was more flame retardant than the ArOPN_2_ and ArOPO_2_ with a P–O–C bond [5]. It is believed that the P–C bond mainly supplied a gas phase radical effect, while P–O–C promoted the char-forming process in the condensed phase. As for EP/polysulfone composites, the gas phase radical effect seems to play a more important role than it does in the condensed phase.

**Figure 4:**
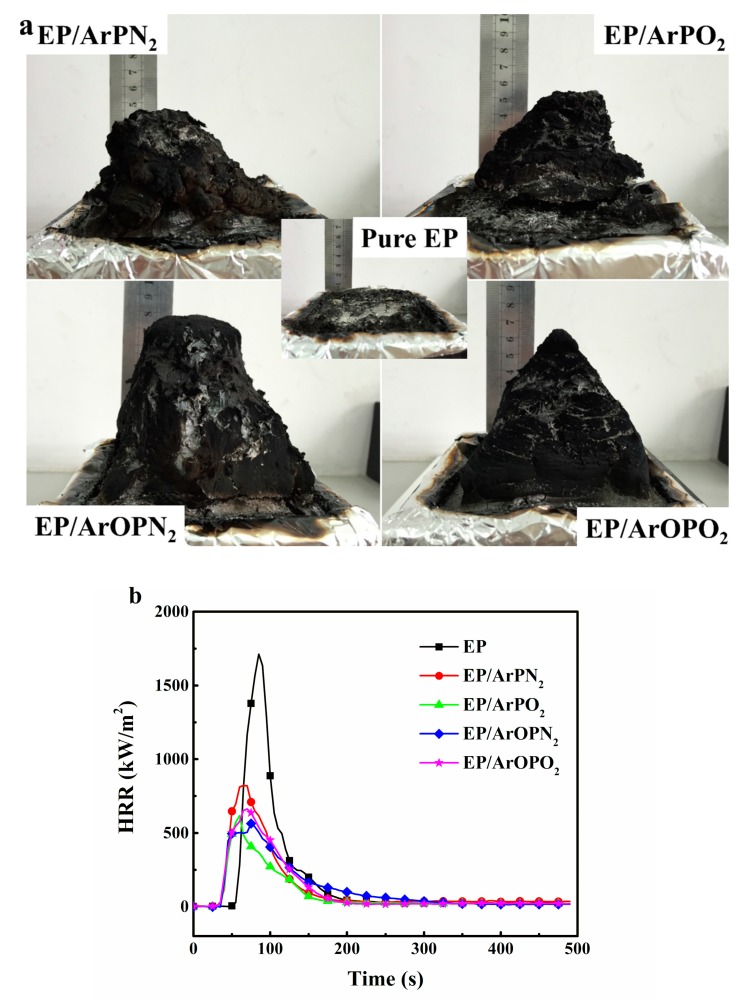
Macroscopic photos of cone calorimeter residue (**a**) and heat release rate (HRR) curves of EP and EP/polysulfone composites (**b**).

Cone calorimeters are widely used to evaluate many important parameters in real fire scenarios, such as the peak heat release rate (PHRR), total heat release (THR), peak smoke production rate (PSPR), total smoke production (TSP), and total carbon monoxide release (TCOR). Figure 4 presents macroscopic photos of residual char and the heat release rate (HRR) curves of EP composites. The characteristic results of all cone results are summarized in Table 4. Pure EP burned quickly and exhibited a limited amount of char. For EP/polysulfone composites, thick and connected three-dimensional residual char was present after cone calorimeter tests. All polysulfones showed a strong char formation effect on the epoxy resins during combustion. The improved intumescent char layer of EP/polysulfone composites acted as an insulating barrier and blocked the exchange of heat, oxygen, and flammable gases between the EP matrix and fire zone.

In the cone calorimeter tests, the addition of polysulfones preserves more mass of the EP composites. The order of the char yields of the EP composites was as follows: EP/ArPO_2_ < EP/ArOPO_2_ < EP/ArPN_2_ < EP/ArOPN_2_. It is noted that polysulfone with a P–O–C bond played a more important role in the char-forming process of EP than polysulfone with a P–C bond. Meanwhile, higher char yields for EP/ArPN_2_ and EP/ArOPN_2_ were observed, which is illustrated by the fact that the degradation gases from the imino group promotes the formation of intumescent char layers.

The time to ignition (TTI) obtained in the cone calorimeter shows differences in the ignition behavior of the various samples. It is in the following order: EP/ArPN_2_ < EP/ArOPN_2_ ≤ EP/ArOPO_2_ < EP/ArPO_2_ < EP. The early TTI for EP/polysulfone composites was associated with the catalysis effect of polysulfones in EP. This agreed well with the TGA experiments. For EP/polysulfone composites, an intumescent char layer formed shortly after ignition. With the protective char layer, the HRR and PHRR of EP/polysulfone composites were significantly decreased. After ignition, the HRR of the EP/polysulfone composites peaked rapidly and then decreased to a low value due to the formation of an intumescent char layer. The PHRR for the EP/polysulfone composites is ordered by increasing fire risk: EP/ArOPN_2_ < EP/ArPO_2_ < EP/ArOPO_2_ < EP/ArPN_2_. The THR divided by the total mass loss (THR/TML) is a measure for the effective heat of combustion and the combustion efficiency of the volatiles [16]. The THR/TML ratio for EP/ArPO_2_ and EP/ArPN_2_ showed a lower value in comparison to EP/ArOPO_2_ and EP/ArOPN_2_, respectively. During combustion, volatiles containing phosphorus were released into the flame zone, resulting in flame inhibition. The effect of the polysulfones with a P–C bond was stronger than that of the polysulfones with a P–O–C bond. Moreover, the THR/TML ratio for EP/ArPN_2_ and EP/ArOPN_2_ was higher than that for EP/ArPO_2_ and EP/ArOPO_2_, respectively, which may be associated with the release of nitrogen-containing gases by the imino group in the main chain of polysulfones.

Smoke and carbon monoxide (CO) from combustion bring serious harm to human health. For EP/ArOPN_2_ and EP/AROPO_2_, the P–O–C structure in the side chain of polysulfones promotes the char-forming process and greatly decreases the TSR. As is well known, the enhanced flame retardant performance of EP results in incomplete combustion. The TSR/TML and TCOR/TML ratios increased in the following order: EP/ArOPN_2_ ≤ EP/ArOPO_2_ < EP/ArPN_2_ < EP/ArPO_2_, suggesting that the polysulfones with a P–C bond show a strong flame inhibition effect in gas phase flame retardancy than those with a P–O–C bond.

### 3.3. Gas Phase Mechanisms

**Figure 5:**
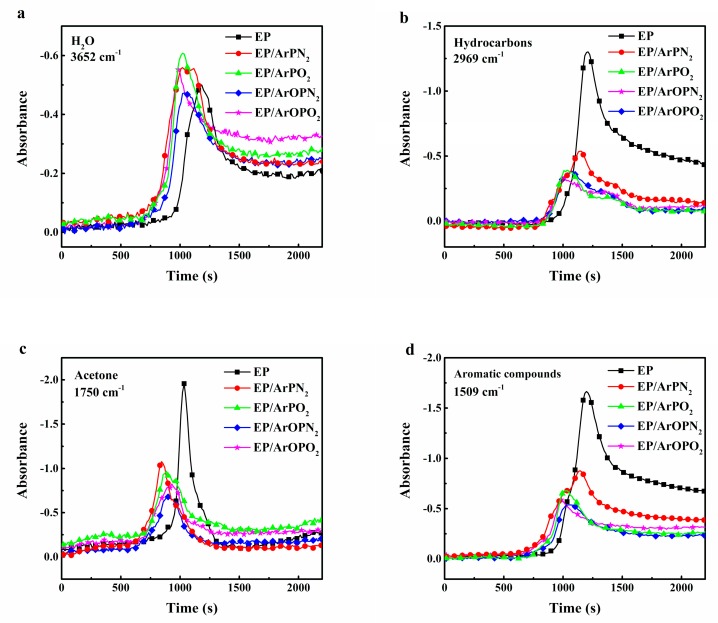
Absorbance of gas products for EP and EP/polysulfone composites vs. time: (**a**) H_2_O; (**b**) hydrocarbons; (**c**) acetone; (**d**) aromatic compounds.

In order to study the gas phase mechanisms of polysulfones, the pyrolysis products during thermal decomposition were analyzed by TGA-FTIR, and the absorbance of the pyrolysis products vs. time is plotted in Figure 5. During the main decomposition step, EP and EP/polysulfone composites released similar products, such as water or phenol (3652 cm^−1^), hydrocarbons (2969 cm^−1^), acetone (1750 cm^−1^), aromatic compounds (1609 and 1509 cm^−1^), and ethers (1259 and 1176 cm^−1^) [17]. With the addition of polysulfone, the pyrolysis products from EP/polysulfone composites were formed earlier than in the case of EP. This can be explained in terms of phosphorus-containing polysulfones catalyzing the thermal decomposition of EP. In addition, the absorbance intensity of flammable gases (acetone and hydrocarbons) was lower than that for EP. Consequently, the addition of phosphorus-containing polysulfones reduced the release of flammable gases.

### 3.4. Condensed Phase Mechanisms

**Figure 6:**
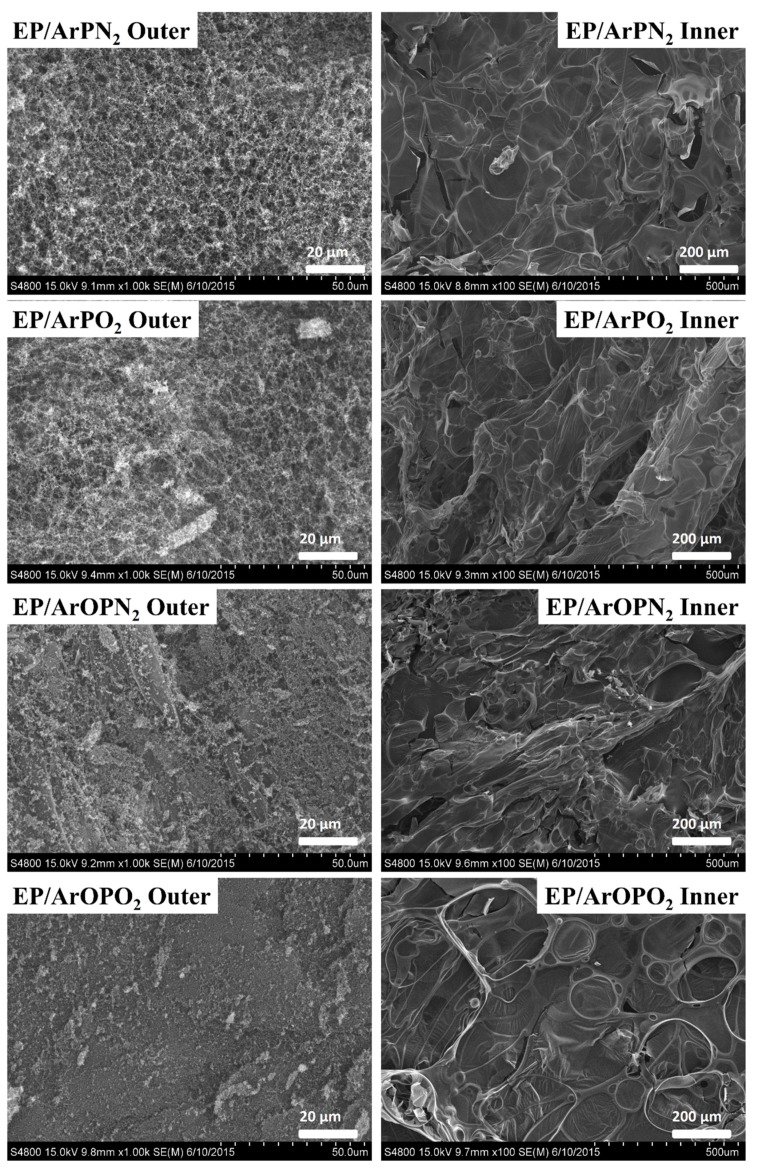
SEM images of surface for residual char of pure EP and EP/polysulfone composites.

As is well known, the residual char plays a crucial role in enhancing the flame retardant performance of polymer composites. Figure 6 presents the SEM images of the outer surface and inner surface of residual chars obtained from the CONE tests. A continuous char layer can be observed on the outer surface of the residual char for all samples. As for the inner surface of residual char, it can be seen that there are many bubble-like sheets, which were caused by the pyrolysis gases in the molten polymer during combustion. As for EP/ArOPN_2_ and EP/ArOPO_2_, the outer surface of residual chars was denser than the surfaces of EP/ArPN_2_ and EP/ArPO_2_, because P–O–C mainly acted in the condensed phase and promoted the char-forming process, while P–C acted both in the condensed phase and the gas phase, and it was easy for the porous char layers to release phosphorus-containing gases. 

To further understand the flame retardant mechanisms, the chemical components on the surface of the residual chars were investigated by XPS spectroscopy, and the results are summarized in Table 4. With the addition of four polysulfones, the content of oxygen and sulfur in the outer residual char of EP/polysulfone composites were much higher than that of pure EP, corresponding to a higher thermal oxidation capacity of residual char. In the case of EP/ArOPN_2_ and EP/ArOPO_2_, a high atom percent of phosphorus, nitrogen, and sulfur indicated that more P-, N-, and S-containing compounds were maintained in the outer surface of residual char. These compounds in the residual char could promote the thermal stability of the char layer and catalyze the char formation. However, it is noted that the percent of phosphorus was very low in the inner surface of EP/ArPN_2_ and EP/ArPO_2_, suggesting that P–C tended to produce phosphorus-containing pyrolysis gases and acted as a free radical in the gas phase. In summary, the P–O–C bond in the EP/polysulfone composites effectively promoted the char forming process better than the P–C bond did, resulting in a more condensed char layer. The P–C bond in the EP/polysulfone composites had a stronger flame inhibition effect in the gas phase than did the P–O–C bond.

### 3.5. Thermal Degradation Kinetics

To better understand the decomposition route of the EP/polysulfone composites, TGA was used to estimate the kinetic parameters of the degradation processes. Flynn–Wall–Ozawa methods (FWO) are widely used to calculate the parameters of the flame retardant composites, such as *E*_a_ (activation energies), *n* (apparent reaction order), and *A* (pre-exponential factor) [18]. The FWO method, using Dolye’s approximation for the integration, is expressed as

$$\log\beta =-\frac{0.457E_{a}}{RT}+\left\{\log\left[\frac{AE_{a}}{g\left(a\right)R}\right]-2.315\right\}$$

At a given conversion degree *α*, the plot of log*β* against −1/*T*_p_ makes a fitted straight line with a slope of −0.457 *E*_a_/*R*. The apparent activation energy *E*_a_ at a given *α* value can be calculated from the value of 0.457 *E*_a_/*R*.

**Figure 7:**
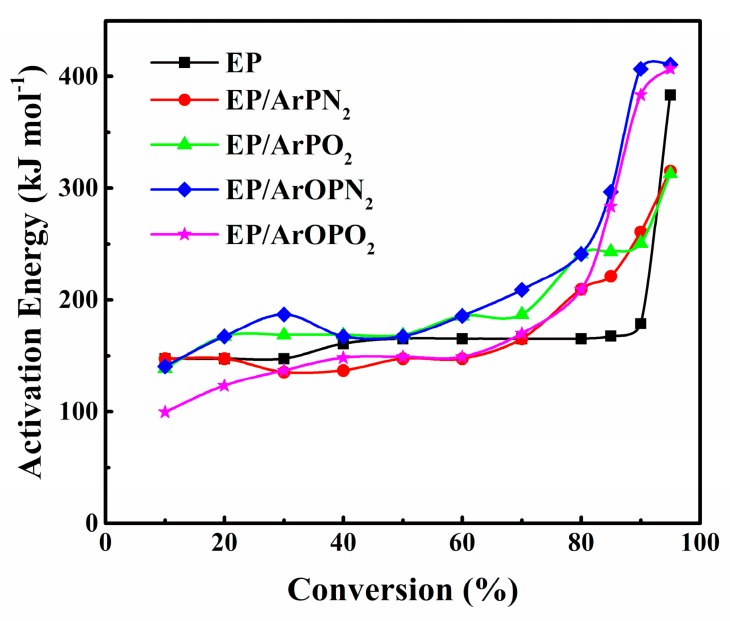
Activation energy curves of pure EP and EP/polysulfone composites.

The calculated *E_a_* values are given in Table 5. The results fitted with the FWO method are shown in Figure 7. As for EP/ArOPO_2_, the calculated *E*_a_ with a value of 99.57 kJ/mol is much lower than that of pure EP (147.37 kJ/mol) and other EP composites, and this was caused by the strong catalyzing effect of P–O–C. With the reaction degree increased from 20% to 40%, EP/ArOPN_2_ had the highest *E*_a_ value, which illustrated that the addition of ArOPN_2_ could promote the formation of an intumescent char layer and enhance the thermal stability of the char layer. This is also shown by the highest char layer of EP/ArOPN_2_ in the CONE tests. When *α* rises to 70~100%, the *E*_a_ value of EP/ArOPN_2_ and EP/ArOPO_2_ are the highest among all EP composites, suggesting that a P–O–C bond in the side chain improves the thermal stability of EP at high temperatures.

As for EP/ArPN_2_ and EP/ArPO_2_, the *E*_a_ values were similar to pure EP when *α* was lower than 70%. When the conversion rate increased to 70~85%, their *E*_a_ values were higher than those of pure EP, which means much more energy was needed to promote the decomposition process. However, when *α* reached 90%, the *E*_a_ values for the two composites were even lower than pure EP, which indicated a lower thermal stability of residual char. The residual char of EP/ArPN_2_ and EP/ArPO_2_ was beneficial for the release of phosphorus-containing pyrolysis gases during the fire. This is in agreement with the porous carbon layer observed from the CONE tests.

## 4. Conclusions

The thermal properties and flame retardancy of four polysulfones (ArPN_2_, ArPO_2_, ArOPN_2_, and ArOPO_2_) and their EP composites (phosphorus content was maintained at 1.25 wt %) were studied. The addition of all polysulfones showed a slight influence on the *T*_g_ of the EP, and the EP composites maintained good stability. All the polysulfones enhanced the thermal stability of the EP and showed a strong intumescent char formation effect on the EP. The improved intumescent char layer during the combustion of EP/polysulfone composites acted as an insulating barrier to block the exchange of heat, oxygen, and flammable gases between the EP matrix and the fire zone, and the PHRR and THR were both greatly reduced. UL-94 vertical burning tests and cone calorimeter tests indicated that the polysulfones with a P–C bond showed a higher flame retardancy than those with a P–O–C bond. TGA and cone calorimeter tests illustrated that the polysulfones containing a P–O–C bond showed a stronger char forming effect on the EP composites than those with a P–C bond, which was mainly responsible for the flame retardant effect in the condensed phase. This work provides a novel method for the design of valuable flame retardant EP used in high temperature environments.

## Figures and Tables

**Table 1:** Compositions and vertical burning tests results of all epoxy composites.

Samples	DGEBA (g)	m-PDA (g)	Polysulfone (g)	P (wt %)	UL-94 (3.2 mm)
EP	50.00	6.00	0	0	No rating
EP/ArPN_2_	50.00	6.00	9.88	1.25	V-0
EP/ArPO_2_	50.00	6.00	9.88	1.25	V-1
EP/ArOPN_2_	50.00	6.00	10.35	1.25	No rating
EP/ArOPO_2_	50.00	6.00	10.35	1.25	No rating

**Table 2:** Thermal properties of epoxy (EP) and EP/polysulfone composites.

Sample	*T*_g_ (°C)	*T*_onset_ (°C)	*T*_max_ (°C)	Char *^a^* Exp./Calcd.
EP	147.8	369	394	12.7/--
EP/ArPN_2_	146.1	317	339,373	21.7/18.0
EP/ArPO_2_	150.7	317	364	24.9/16.4
EP/ArOPN_2_	134.0	313	365	25.2/17.5
EP/ArOPO_2_	145.5	310	365	27.5/17.0

*^a^* Char yield at 700 °C.

**Table 3:** Cone calorimetric data for EP and EP/polysulfone composites at 50 kW·m^−2^.

Sample	TTI (s)	PHRR (kW·m^−2^)	THR (MJ·m^−2^)	TML (%)	TSR (m^2^)	THR/TML (MJ·m^−2^·g^−1^)	TSR/TML (g^−1^)	TCOR/TML (g·g^−1^)
EP	50	1712	83.7	93.0	22.4	3.85	117	0.11
EP/ArPN_2_	29	847	61.5	80.2	18.3	3.27	110	0.15
EP/ArPO_2_	32	608	42.7	82.7	19.2	2.31	117	0.18
EP/ArOPN_2_	30	546	59.4	77.1	11.4	3.42	75	0.14
EP/ArOPO_2_	30	726	55.3	80.8	14.8	3.09	94	0.14

**Table 4:** X-ray photoelectron spectroscopy (XPS) results of the residual char of EP and EP/polysulfone composites.

Sample	C (% *^a^*)	N (%)	O (%)	P (%)	S (%)
EP	82.82	3.22	13.77	0.10	0.09
EP/ArPN_2_ Inner	82.49	1.10	16.10	0.13	0.18
EP/ArPN_2_ Outer	79.19	3.16	16.65	0.84	0.17
EP/ArPO_2_ Inner	81.80	1.11	16.74	0.14	0.21
EP/ArPO_2_ Outer	75.52	2.88	19.28	2.05	0.26
EP/ArOPN_2_ Inner	80.87	1.09	17.52	0.28	0.24
EP/ArOPN_2_ Outer	71.34	5.53	19.78	2.92	0.43
EP/ArOPO_2_ Inner	80.92	3.26	14.35	1.24	0.23
EP/ArOPO_2_ Outer	72.17	4.50	19.98	2.87	0.48

*^a^* Atomic concentration.

**Table 5:** Calculated *E*_a_ at various conversions using the FWO method for EP/polysulfone composites.

α	EP		EP/ArPN_2_		EP/ArPO_2_		EP/ArOPN_2_		EP/ArOPO_2_	
	*E*_a_ (kJ/mol)	*r*	*E*_a_ (kJ/mol)	*r*	*E*_a_ (kJ/mol)	*r*	*E*_a_ (kJ/mol)	*r*	*E*_a_ (kJ/mol)	*r*
0.10	147.37	0.9967	147.37	0.9967	138.74	0.9716	140.61	0.9816	99.57	0.9145
0.20	147.37	0.9967	147.37	0.9967	167.26	0.9593	167.26	0.9593	123.07	0.9210
0.30	147.37	0.9967	135.22	0.9907	168.51	0.9884	187.00	0.9665	136.91	0.9220
0.40	160.52	0.9974	136.91	1.0000	168.51	0.9884	167.26	0.9593	148.01	0.9586
0.50	165.05	0.9959	147.37	0.9967	168.51	0.9884	167.26	0.9593	148.74	0.9189
0.60	165.05	0.9959	147.37	0.9967	185.75	0.9876	185.75	0.9876	148.74	0.9189
0.70	165.05	0.9959	165.05	0.9959	187.00	0.9665	209.03	0.9650	169.86	0.9215
0.80	165.05	0.9959	209.40	0.9956	240.97	0.9757	240.97	0.9757	209.03	0.9650
0.85	167.34	0.9949	221.17	0.9962	243.09	0.9370	296.63	0.9657	283.60	0.9604
0.90	178.58	0.9836	260.79	0.9636	250.73	0.8971	406.83	0.9740	383.36	0.9849
0.95	383.36	0.9849	315.60	0.9846	312.94	0.9335	410.74	0.9220	406.83	0.9740
